# MarVis-Pathway: integrative and exploratory pathway analysis of non-targeted metabolomics data

**DOI:** 10.1007/s11306-014-0734-y

**Published:** 2014-10-10

**Authors:** Alexander Kaever, Manuel Landesfeind, Kirstin Feussner, Alina Mosblech, Ingo Heilmann, Burkhard Morgenstern, Ivo Feussner, Peter Meinicke

**Affiliations:** 1 Department of Bioinformatics, Institute of Microbiology and Genetics, Georg-August-University Göttingen, Goldschmidtstr. 1, 37077 Göttingen, Germany; 2 Department of Plant Biochemistry, Albrecht-von-Haller-Institute for Plant Sciences, Georg-August-University Göttingen, Justus-von-Liebig-Weg 11, 37077 Göttingen, Germany

**Keywords:** Metabolomics, Metabolic fingerprinting, Mass spectrometry, Metabolic pathways, Set enrichment analysis, Transcriptomics

## Abstract

A central aim in the evaluation of non-targeted metabolomics data is the detection of intensity patterns that differ between experimental conditions as well as the identification of the underlying metabolites and their association with metabolic pathways. In this context, the identification of metabolites based on non-targeted mass spectrometry data is a major bottleneck. In many applications, this identification needs to be guided by expert knowledge and interactive tools for exploratory data analysis can significantly support this process. Additionally, the integration of data from other omics platforms, such as DNA microarray-based transcriptomics, can provide valuable hints and thereby facilitate the identification of metabolites via the reconstruction of related metabolic pathways. We here introduce the MarVis-Pathway tool, which allows the user to identify metabolites by annotation of pathways from cross-omics data. The analysis is supported by an extensive framework for pathway enrichment and meta-analysis. The tool allows the mapping of data set features by ID, name, and accurate mass, and can incorporate information from adduct and isotope correction of mass spectrometry data. MarVis-Pathway was integrated in the MarVis-Suite (http://marvis.gobics.de), which features the seamless highly interactive filtering, combination, clustering, and visualization of omics data sets. The functionality of the new software tool is illustrated using combined mass spectrometry and DNA microarray data. This application confirms jasmonate biosynthesis as important metabolic pathway that is upregulated during the wound response of Arabidopsis plants.

**Electronic supplementary material:**

The online version of this article (doi:10.1007/s11306-014-0734-y) contains supplementary material, which is available to authorized users.

## Introduction

Metabolomics studies (Dunn et al. 2013; Fiehn 2002) aim to identify and characterize all metabolites under specific experimental conditions, such as environmental or genetic perturbations or developmental stages (Tarpley et al. 2005; Nahlik et al. 2010; Watanabe et al. 2013; Bellaire et al. 2013; König et al. 2014). In this field, mass spectrometry (MS) coupled to gas or liquid chromatography (GC/MS and LC/MS) has become a key technology for detection, identification, and quantification of metabolites (Dunn et al. 2005). A typical non-targeted metabolomics experiment can be represented by a high-dimensional data matrix (Dettmer et al. 2007; Meinicke et al. 2008) comprising information on the identity of measured ion species (data set features) and intensities for each feature and sample. These intensities can be used as relative abundance measurements for the comparison of different samples or groups of samples. The features are characterized by means of the mass-to-charge (m/z) ratio, retention time (rt), and the respective intensity profiles. Data sets from other omics technologies, such as DNA microarray or RNA-seq-based transcriptomics (Brown and Botstein 1999; Mortazavi et al. 2008) and MS-based proteomics (Aebersold and Mann 2003), may be represented in a similar way. After preprocessing, the corresponding data set features, e.g. DNA microarray spots, can be identified with associated gene, protein, or transcript IDs. Similar to the non-targeted MS data from a metabolomics experiment, where a particular metabolite may be represented by multiple features standing for different isotopologues and adducts (Brown et al. 2009; Draper et al. 2009), a transcript may be associated with multiple spots containing specific DNA probes. The typical workflow in the analysis of omics data involves several steps for the identification and characterization of data set features that are relevant in a particular context. For this purpose, replicate samples for each experimental condition are statistically evaluated in order to identify features which show significant differences (Dudoit et al. 2002; Sugimoto et al. 2012; Kaever et al. 2012). In many applications, e.g. when analyzing time series, the experiments comprise more than two conditions and preprocessing results in large data sets of complex multivariate intensity profiles.

After detection of features, which significantly differ between conditions, the filtered data set can be analyzed by means of exploratory multivariate methods, such as clustering algorithms, principal, or independent component analysis (Eisen et al. 1998; Dettmer et al. 2007; Gürdeniz et al. 2013; Meinicke et al. 2008; Wijetunge et al. 2013) in order to identify prominent intensity patterns. Finally, annotations, e.g. in terms of metabolic pathways, may be used to explain or characterize particular groups of features in a functional context (Dahlquist et al. 2002; Suhre and Schmitt-Kopplin 2008). Pathway maps from public databases, such as the Kyoto Encyclopedia of Genes and Genomes (KEGG) (Kanehisa et al. 2012) and BioCyc (Caspi et al. 2012), contain information about metabolic reactions as well as the associated enzymes, genes, and metabolites, and can therefore interconnect almost all omics fields (Arakawa et al. 2005; Wägele et al. 2012). While the mapping of gene and protein IDs is in most cases straightforward, m/z ratios from non-targeted metabolomics experiments cannot be directly mapped to entries in the corresponding databases and the identification of metabolites is a major bottleneck in such experiments (Dunn et al. 2013; Scalbert et al. 2009). A common approach is to calculate putative monoisotopic masses and molecular formulas for all MS data set features and match these with known metabolites (Brown et al. 2011; Kuhl et al. 2012; Kaever et al. 2012; Lee et al. 2013). In order to identify relevant pathways, a popular approach is the Gene/Metabolite Set Enrichment Analysis (G/M SEA) and Over-Representation Analysis (ORA) (Subramanian et al. 2005; Xia and Wishart 2010; Persicke et al. 2012; Khatri et al. 2012), where pathways are represented as sets of entries, e.g. metabolites in MSEA. The enrichment analysis aims to detect pathways which are enriched in significant or high-ranked features mapped to corresponding entries.

For the analysis of MS-derived metabolomics data, several web-based platforms have been published that cover all steps from preprocessing, data set management, statistical analysis, mapping of features to metabolic pathways, and enrichment analysis (Kessler et al. 2013; Xia et al. 2012; Kastenmüller et al. 2011; Wägele et al. 2012). Only recently, the stand-alone software MetaboNexus (Huang et al. 2014), which combines a workflow similar to the web-based platforms with the manual selection and database query of MS features, was introduced. MetaboNexus provides a browser-based user interface, but the analysis is performed on the local machine and without requiring the upload of data sets to a web server. In the context of DNA microarray analysis, software tools and libraries which allow the exploratory data analysis by means of cluster algorithms are available (Eisen et al. 1998; Saldanha 2004; Sturn et al. 2002; Hoon et al. 2004) and the methodology of GSEA and ORA was implemented in multiple packages (Huang et al. 2009; Ackermann and Strimmer 2009; Khatri et al. 2012). Powerful software suites, such as the TM4 platform (Saeed et al. 2003, 2006), allow the interactive and exploratory analysis of microarray data, e.g. the clustering and labeling of transcript profiles, in combination with ORA. In order to combine and integrate results from different omics platforms, many tools which focus on visualization, e.g. based on metabolic pathways, have been proposed (Gehlenborg et al. 2010; Thimm et al. 2004; Junker et al. 2006; Neuweger et al. 2009). Different platforms for the network-based visualization and analysis of metabolomics and transcriptomics data have been introduced (Gao et al. 2010; Landesfeind et al. 2014; Posma et al. 2014). The Cytoscape (Shannon et al. 2003) plug-in Metscape (Gao et al. 2010), for example, allows the extraction of pathway-specific subnetworks, the coloring of nodes according to intensities, and the animation of different condition-specific snapshots.

The MarVis-Suite tools (Kaever et al. 2009, 2012) were introduced for the extraction, clustering, and visualization of metabolic markers from data originating from non-targeted experiments. The MarVis-Suite thereby combines functionalities of previously described tools and platforms with the focus on three main themes: It provides highly interactive desktop user interfaces, e.g. for interactive inspection of data clusters, thus integrating the user’s expert knowledge instead of generating static heatmap figures. For the analysis of data from non-targeted MS experiments, specialized functions are provided. These tools are combined with more general functions that allow the straightforward integration of data sets from other omics platforms. In particular, the MarVis-Cluster interface provides a robust clustering based on one-dimensional self-organizing maps (1D-SOMs) (Meinicke et al. 2008), that is interactively used to investigate intensity patterns for a large number of multivariate feature profiles. Additionally, the MarVis-Filter interface features the adduct and isotope correction, filtering, and combination of multiple data sets, e.g. derived from positive and negative ionization mode. Several tools of the MarVis-Suite have been successfully applied for the identification of metabolite markers relevant in plant-pathogen-interaction (Djamei et al. 2011; Floerl et al. 2012; König et al. 2014) as well as for the characterization of mutants in lipid metabolism of Arabidopsis (König et al. 2012) and the COP9 signalosome of Aspergillus (Nahlik et al. 2010; Gerke et al. 2012).

**Fig. 1:**
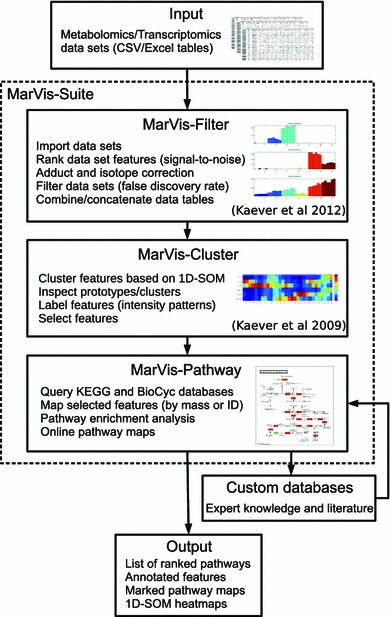
Interactive workflow of data analysis within the MarVis-Suite

In order to identify data set features in a functional context, we introduce the MarVis-Pathway tool, which allows the annotation and analysis of organism-specific pathways from the KEGG and BioCyc database collections in combination with an SEA meta-analysis framework for multi-omics data sets (Kaever et al. 2014). The mapping of features to database entries is based on the matching of IDs, names, or accurate masses. MarVis-Pathway thereby completes the MarVis-Suite pipeline by providing a knowledge-based interpretation of results from explorative data analysis (see Fig. 1 for an overview on the interactive workflow). In addition, we introduce a signal-to-noise ratio-based ranking and filtering method for the MarVis-Filter tool, which features the statistical analysis of heterogeneous omics data based on minimal assumptions and which can be easily used for exploratory data analysis by modifying the signal definition. The proposed methods and tools are applied to data sets combining LC/MS with DNA microarray data in the context of a cross-omics study on the wound response of Arabidopsis plants, which represents a well-established model system. We show that the strength of MarVis-Pathway lies in the enhancement of analysis and interpretation of non-targeted LC/MS data sets in combination with transcriptomics data.

## Materials and methods

### Availability

Installation packages for the MarVis-Suite including MarVis-Pathway and a detailed handbook are available on the project homepage http://marvis.gobics.de. Data sets are available as comma separated values (CSV) files. Additionally, a detailed protocol of the corresponding data analysis within the MarVis-Suite and project files which can be loaded directly into the MarVis-Suite interfaces (Load project function in the File menu) are provided.

### Study, data sets, and preprocessing

The study investigates the wound reaction of *Arabidopsis thaliana* (ecotype Columbia-0) wild type (wt) and jasmonate-deficient *dde*2-2 mutant plants (Malek et al. 2002) in a time course (control, 0.5 hours post wounding (hpw), 2 hpw) and comprises four metabolomics LC/MS and one transcriptomics DNA microarray data set generated from the same biological samples (see Table 1). The study was performed as described in (Mosblech et al. 2008; Meinicke et al. 2008; Kaever et al. 2012). For each of six experimental conditions (wt: control, wt: 0.5 hpw, wt: 2 hpw, *dde*2-2: control, *dde*2-2: 0.5 hpw, *dde*2-2: 2 hpw), three biological replicate samples were analyzed with two platforms: The four metabolomics data sets derive from Ultra Performance Liquid Chromatography (UPLC) coupled to a Time-Of-Flight (TOF) MS analysis of the non-polar and polar extraction phases in positive and negative ionization mode, respectively (see Table 1). For each sample, two UPLC TOF-MS runs (technical replicates) were performed, which resulted in six replicates per experimental condition. Data processing of the raw UPLC TOF-MS data (peak picking, peak alignment, and deisotoping) was performed with the MarkerLynx Application Manager for the MassLynx software (Waters Corporation, Milford, USA). For the DNA microarray analysis, the Agilent-021169 Arabidopsis 4 Oligo Microarray (V4) platform was used. Spots without gene assignment were left out and the expression values were quantile-normalized.

**Table 1:** Overview on data sets used for the integrative metabolome and transcriptome study of wild type and jasmonate-deficient *dde*2-2 mutant plants in a time course of 0, 0.5, and 2 hours post wounding (6 conditions)

Data set label	Platform	Conditions/samples per condition	Extraction phase	Ionization mode	Features	Filtered features
M1	UPLC TOF-MS	6/3^a^	Non-polar	Negative	2,272	316
M2	UPLC TOF-MS	6/3^a^	Non-polar	Positive	5,980	313
M3	UPLC TOF-MS	6/3^a^	Polar	Negative	4,023	161
M4	UPLC TOF-MS	6/3^a^	Polar	Positive	10,421	234
T1	DNA microarray	6/3	–	–	38,825	2,809

The number of data set features/variables corresponds to the number of different ion species detected in MS analysis and the number of microarray spots (after discarding spots which were not assigned to a gene), respectively. The last column shows the number of retained features after signal-to-noise filtering ($FDR < 0.05$ in random permutation test, see Sect. 2.3)

^a^The metabolomics data sets comprise two technical replicates per sample.

### MarVis filter: data import, adduct correction, signal-to-noise filtering, and combination of data sets

The metabolomics and transcriptomics data sets were consecutively imported in MarVis-Filter (Kaever et al. 2012; see also Raw data import function in the MarVis-Suite handbook) and processed (see Table 1; Fig. 1). In order to calculate accurate monoisotopic masses for all ion features in the MS data sets, the m/z values were corrected in MarVis-Filter based on different sets of rules for positive and negative ionization mode (mass tolerance 0.01 Da, rt tolerance 0.05 min) as described in (Kaever et al. 2012). The features of each of the five data sets were filtered according to a signal-to-noise ratio (SNR) (He and Zhou 2008) in combination with 1000 random permutations of sample labels (assignments of samples to conditions) and a false discovery rate (FDR) (Benjamini and Hochberg 1995) threshold of 0.05 (see MarVis-Suite handbook), similar to the Significance Analysis of Microarrays (SAM) method introduced by Tusher et al. (2001). As part of the SNR calculation for each feature, the signal was defined as difference between the maximum and minimum average condition-specific intensity and the noise term was calculated as pooled sample standard deviation of intensity values over all conditions. For the metabolomics data sets, which contain two technical replicates per biological sample, the FDRs were estimated by randomly permuting only the biological samples (see labeling of dependent replicates in the MarVis-Suite handbook). The technical replicates were always assigned to the condition label of the corresponding sample. This procedure allows to utilize the technical variation in the SNR score calculation without assuming independence of technical replicates, which usually show a high dependence. The intensities are not assumed to follow a specific distribution, e.g. the normal or log-normal distribution, which considerably extends the range of application and allows to filter heterogeneous data sets, e.g. metabolome and transcriptome data, within the same framework. Table 1 gives an overview on the number of features after filtering. For a customized SNR (see MarVis-Suite handbook), the signal may also be defined as the difference of the maximum/minimum/mean of average intensities for two subsets of conditions, e.g. comparing the maximum of condition 2 and 3 with the maximum over all other (control) conditions. Each filtered data set was stored in the MarVis-Filter clipboard. Finally, all filtered metabolomics and transcriptomics data sets were combined by concatenating the corresponding data tables (see MarVis-Suite documentation).

### MarVis-Cluster: clustering, visualization, selection, and labeling of data set features

The combined data set was clustered and visualized in MarVis-Cluster (Kaever et al. 2009; see also Goto MarVis-Cluster function in the MarVis-Suite handbook) using 30 prototypes/clusters for the training of the 1D-SOM. For clustering, the replicate intensities per condition and feature were averaged (arithmetic mean) and the resulting profile was normalized to unit Euclidean length. For each cluster, the proportion of metabolomics and transcriptomics features was visualized (see the Label barplot function in the MarVis-Suite handbook). In order to label features which show higher intensities in the wt wounding-specific conditions compared to *dde*2-2, all features were selected and the selection was reduced by means of a customized SNR (see Sect. 2.3 and the MarVis-Suite handbook). For this purpose, the signal was defined as the difference between the maximum of the average intensities for condition 2 and 3 (wt: 0.5 hpw and wt: 2 hpw) and the maximum of all other conditions (wt: control and all conditions associated with *dde*2-2). The selection was reduced to all features with a ratio higher than 2 (1506 features) and labeled (’wt’). For the functional analysis in MarVis-Pathway, all features (labeled and unlabeled) were then selected (see Goto MarVis-Pathway function in the MarVis-Suite handbook).

### MarVis-Pathway: database query, pathway enrichment, and meta-analysis

#### Pathway databases and feature mapping

MarVis-Pathway implements pathway databases from the KEGG and the BioCyc collection (Kanehisa et al. (2012); Caspi et al. (2012); see also Fig. 1). The included KEGG collection (KEGG FTP Release Dec 9, 2013, http://www.kegg.jp) contains one reference and about 3,000 organism-specific databases. The included BioCyc collection (biocyc-17.5, http://biocyc.org) provides about the same number of organism-specific databases and one reference database (MetaCyc). Each KEGG reference pathway is associated with a number of compound, EC (Enzyme Commission), and KO (KEGG ORTHOLOGY) IDs and names. Each MetaCyc reference pathway variant is associated with a number of compound and EC IDs/names. For all compounds in the databases, the monoisotopic masses were calculated based on the molecular formula. In case of the organism-specific databases, the pathways are associated with compound IDs, names, and masses and gene IDs/names instead of the EC and KO numbers. Additionally, customized databases may be loaded from comma separated values (CSV) files (see the MarVis-Suite handbook for details).

The features of the combined data set were mapped to metabolite and gene entries in the *A. thaliana*-specific pathways from KEGG and AraCyc (Mueller et al. 2003), which is part of the BioCyc database collection. The mapping of the features from the metabolomics data sets to metabolite entries was based on the corrected accurate masses (see Sect. 2.3) and a tolerance of 0.01 Dalton. The transcriptomics features were mapped to gene entries using the corresponding IDs.

#### Pathway enrichment analysis

For statistical analysis of pathways with matched entries, MarVis-Pathway provides an extensive framework for (Gene/Metabolite) Set Enrichment Analysis (SEA) (Subramanian et al. 2005; Xia and Wishart 2010; Huang et al. 2009). The SEA framework in MarVis-Pathway offers three different types of enrichment analysis: Entry-based, marker/feature-based, and sample-based analysis. In the first case, the number of entries in a pathway matched by the selected features (in MarVis-Filter or MarVis-Cluster) in comparison to the number of entries which could be matched over all pathways is evaluated based on a hypergeometric distribution, similar to the ORA approach (Khatri et al. 2012) introduced by Draghici et al. (2003) and Hosack et al. (2003). When analyzing MS data sets, the metabolite entries are clustered according to their mass before performing the hypergeometric test in order to reduce the systematic dependence of database entries. In case of the marker/feature-based SEA, the analysis is based on the ranks of features (as calculated in MarVis-Filter) which match entries in a particular pathway, assuming independence of features. For statistical evaluation, a static or iterative hypergeometric test (Breitling et al. 2004), a rank-sum, or a Kolmogorov-Smirnov test is utilized. The method is able to incorporate information from adduct and isotope corrections performed in MarVis-Filter. In case of the sample-based SEA, the analysis is based on the ranks of features and a rank-sum or Kolmogorov–Smirnov test statistic which is recalculated for a large number of random permutations of sample condition labels, similar to the original GSEA method (Subramanian et al. 2005). For (re-)ranking, the SNR function is used. This method does not depend on the assumption of independent features or independent database entries but requires a sufficiently high number of replicate samples and considerably more computing time in comparison to the first two methods. As for the SNR permutation test, the labels of technical replicates of the same biological sample may be permuted together. The introduced methods for marker/feature-based and sample-based enrichment analysis use concepts of the Functional Class Scoring (FCS) approaches (Khatri et al. 2012). A detailed description of the implemented types of enrichment analysis can be found in the MarVis-Suite handbook.

#### Meta-analysis of multiple data sets

MarVis-Pathway offers a framework for the joint (entry, marker/feature, or sample-based) SEA of combined data sets. For this purpose, the pathway-specific *p*-values are first calculated for each data set separately in order to account for data set-specific properties, such as the number of features. Then, the *p*-values are merged per pathway in a meta-analysis (Kaever et al. 2014; Shen and Tseng 2010; Whitlock 2005) using Fisher’s (Fisher 1925) or Stouffer’s method (Stouffer et al. 1949) for independent data sets. In case a sample-based enrichment analysis is performed, biological samples in different data sets may be linked and the condition labels are permuted together, e.g. a particular sample is always assigned the same condition label in all linked data sets. The linking option may also be combined with technical replicates belonging to independent biological samples. Finally, the FDRs are calculated (Benjamini and Hochberg 1995) based on the meta-*p*-values. In case a random permutation test is performed, the observed meta-*p*-value for a particular pathway is compared to the meta-*p*-values obtained for all pathways and all random permutations and the corresponding FDR is estimated (Tusher et al. 2001).

In order to identify relevant pathways in the study, entry, marker/feature, and sample-based enrichment analyses were performed. Global pathways with more than 500 associated entries, such as KEGG’s unspecific metabolic pathways map, were left out in this analysis. In case of the entry and marker/feature-based analysis, the *p*-values were calculated based on a hypergeometric test and the initial filtering of the data sets (see Sect. 2.3). For meta-analysis, Fisher’s method was used. In case of the sample-based analysis, a Kolmogorov-Smirnov test in combination with Fisher’s method was used. The obtained meta-*p*-values were recalculated for 1,000 random permutations of sample labels, linking technical replicates within the data sets M1 to M4 and samples over all data sets.

### MS/MS analysis

For unequivocal identification of metabolites, MS/MS spectra of MS features mapped to jasmonic acid (JA), jasmonoyl isoleucine (JA-Ile), 11/12-Hydroxy-JA, 12-Hydroxy-JA-Ile, and 12-Carboxy-JA-Ile were obtained by LC 1290 Infinity (Agilent Technologies, Santa Clara, CA, USA) coupled with a 6540 UHD Accurate-Mass Q-TOF-MS instrument (Agilent Technologies, Santa Clara, CA, USA) with Dual Jet Stream Technology as electrospray ionization (ESI) source (see Supplementary material 4). The analysis was performed in the negative ESI mode with minor modifications as described by Floerl et al. (2012).

## Results and discussion

The plant’s response to wounding is part of the defense against insects and is mainly regulated by the isoleucine conjugate of jasmonic acid JA-Ile (Howe and Jander 2008; Mosblech et al. 2009; Wasternack and Hause 2007; Wu and Baldwin 2010). During recent years, the corresponding defense pathway has been analyzed in detail in Arabidopsis and Tobacco. In the model plant Arabidopsis so far the focus was on transcriptomics and proteomics experiments, comparing wounded wild type plants with JA-Ile biosynthesis or perception mutants (Stintzi et al. 2001; Reymond et al. 2004; Gfeller et al. 2011). Therefore, we used the JA-Ile-dependent wound response of Arabidopsis as an ideal experimental background to evaluate the functionality of MarVis-Pathway and the new MarVis-Suite.

### Intensity profile clustering and visualization provides a convenient overview for combined cross-omics data set

**Fig. 2:**
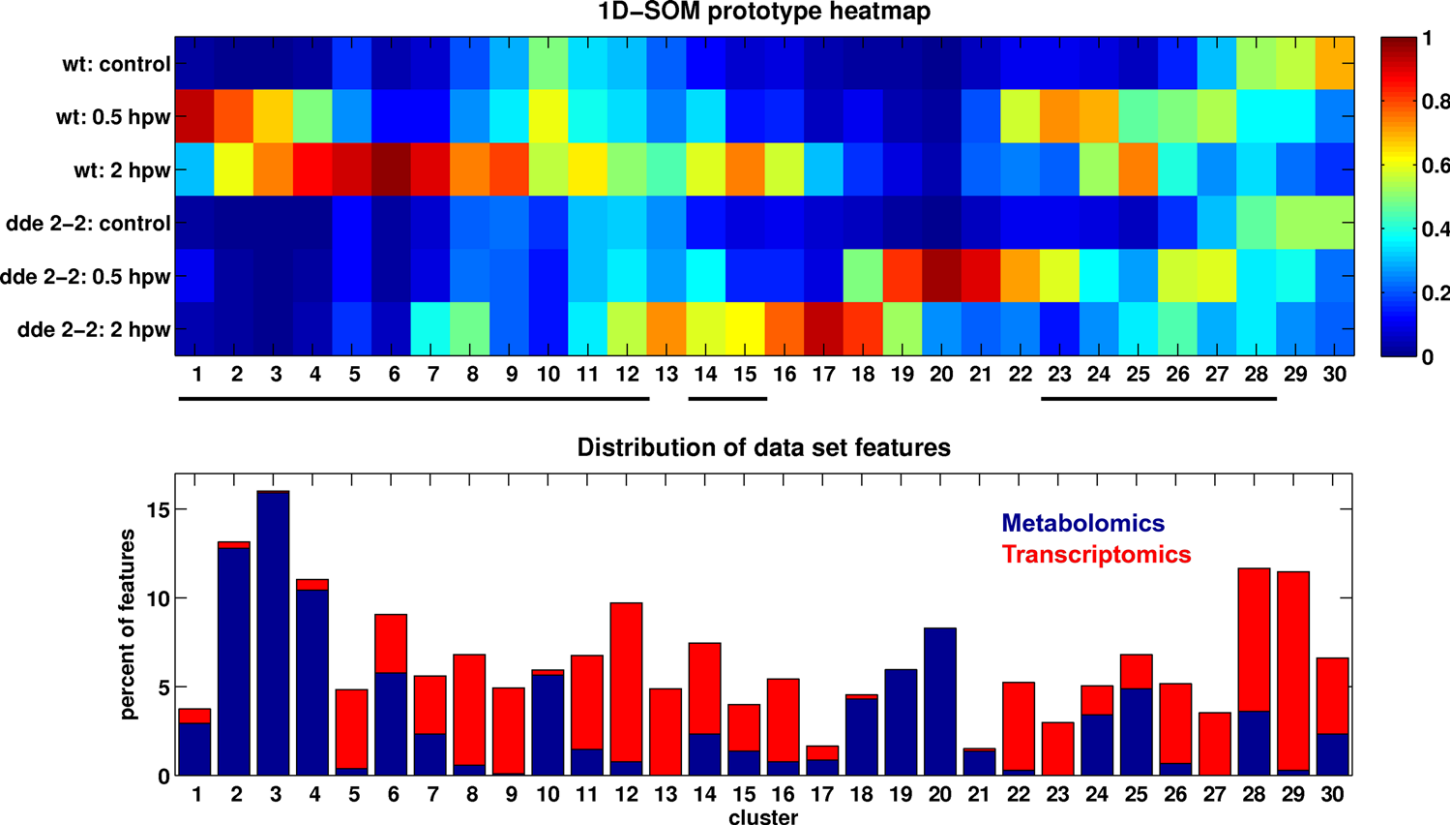
Heatmap of ordered prototype profiles (average cluster profiles) from 1D-SOM clustering (*upper region*) and *stacked bar plot* of the distribution of data set features (*lower region*) for the combined metabolomics and transcriptomics data set. *Blue bars* in the *lower plot* indicate the percentage of features from the metabolomics data sets (ion species) found in the corresponding cluster. *Red bars* show the percentage of transcriptomics features (*microarray spots*), respectively. *Black lines* between the prototype and *bar plot* mark clusters that contain features which were labeled as wt-specific by means of a customized SNR (see Sect. 2.4)

The filtered transcriptomics and four metabolomics data sets were combined in MarVis-Filter (see Sects. 2.2, 2.3) and analyzed in MarVis-Cluster (see Sect. 2.4 and workflow in Fig. 1). Figure 2 shows the heatmap of prototypes (average cluster profiles) and the proportion of metabolomics and transcriptomics features within each cluster. The upper prototype plot provides a convenient overview on prominent intensity patterns and allows to interactively browse the clusters and select features. The first block of clusters (prototype 1–6) represents metabolomics and transcriptomics features with a profile specific for the wound response in wt plants. These features are therefore dependent on the biosynthesis of the signal molecule JA-Ile. However, a closer inspection revealed that also other clusters (e.g. cluster 7 and 8, see Fig. 2) harbor additional features being JA-Ile-dependent and showing a less prominent but significant difference. In order to mark all these wt-specific features in further analysis, they were labeled utilizing a customized SNR (see Sect. 2.4).

An important issue in the context of integrative analysis of metabolomics and transcriptomics time series data is the possible time lag between the different omics levels (Takahashi et al. 2011; Gibon et al. 2006). For example, transcripts may not be translated for a couple of hours resulting in a time shift of corresponding metabolite products. The heatmap visualization (see Fig. 2) supports the interactive analysis of different time frames, e.g. by means of the identification of blocks of clusters representing an early or late wound response (see cluster 1 and 2 or 3–6). However, the introduced functions focus on the visualization and interactive analysis of time-dependent intensity patterns and not on the calculation of time lags between different omics levels.

### MarVis-Pathway facilitates the reconstruction and interactive analysis of metabolic pathways

For a functional interpretation, all metabolomics and transcriptomics features were selected and used for analysis in MarVis-Pathway (see Sect. 2.5). Based on the corrected monoisotopic masses (see Sect. 2.3) and gene IDs, the features were mapped to entries in the *A. thaliana*-specific pathways from KEGG and AraCyc (Mueller et al. 2003).

**Fig. 3:**
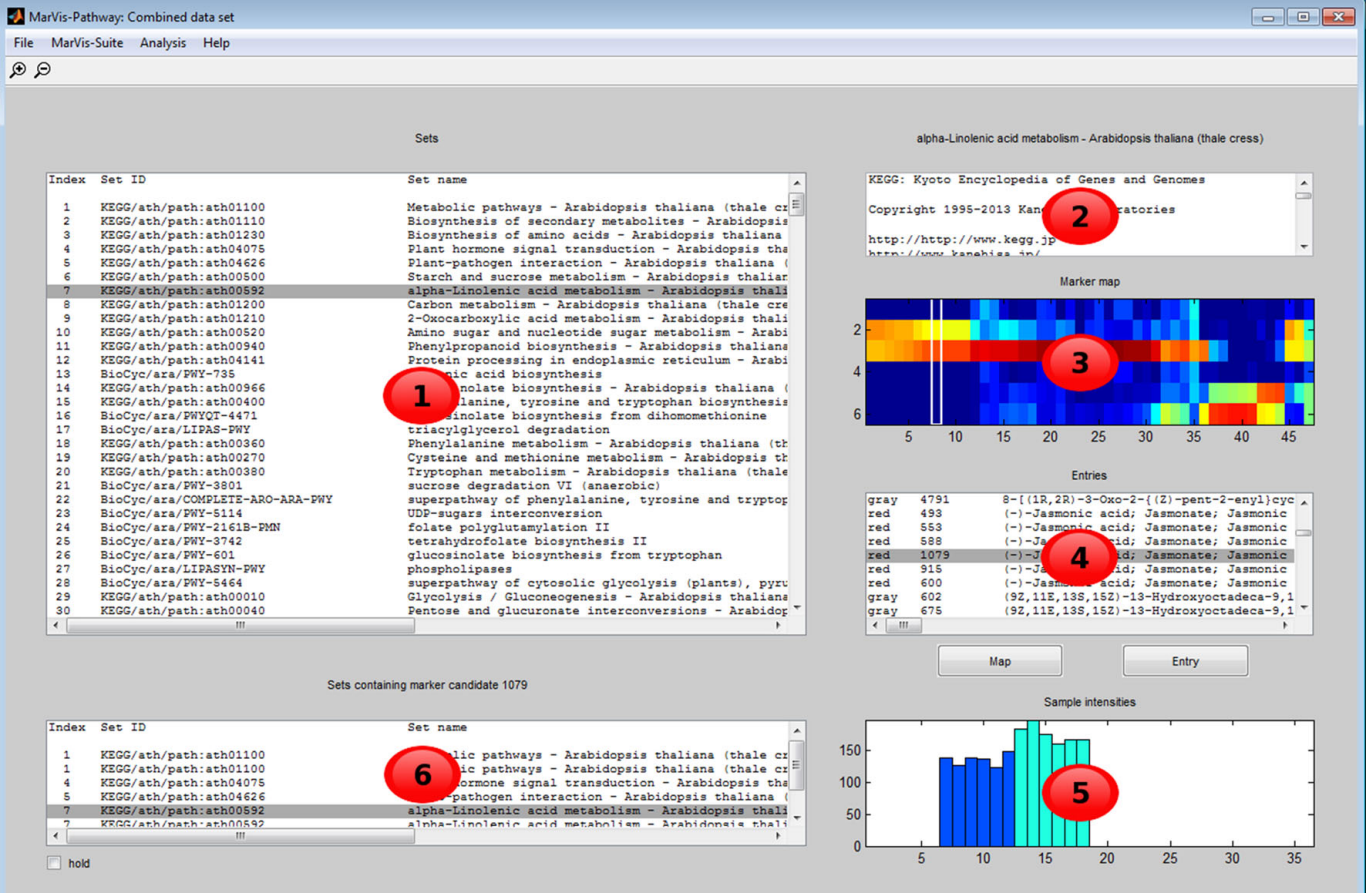
Screenshot of the MarVis-Pathway interface after database query. The pathway list box (area* 1*) contains all matched pathways. The pathway information box (*2*) contains additional information about the flat files used for database construction. The marker profile map (*3*) shows the heatmap of feature profiles which could be mapped to the selected pathway. The entry assignment list box (*4*) contains the assignments of features to entries in the selected pathway. The marker profile plot (*5*) displays the raw intensity profile of the currently selected feature. The related pathways list box (*6*) shows all pathways that contain entries mapped to the currently selected data set feature. Pathways, profiles, and entry assignments can be interactively inspected and selected. Via the Map and Entry button below the assignment list box (*4*), the online resources of the queried databases can be accessed, the marker color of particular entries may be interactively or automatically specified (only for KEGG pathways)

Figure 3 shows a screenshot of MarVis-Pathway after database query together with a short description of the interactive user interface. Pathways which contain matched metabolites or genes can be interactively inspected and selected. For the selected pathway, the averaged and normalized intensity profiles of associated features (see Sect. 2.4) are visualized in a heatmap sorted according to the 1D-SOM order, which allows a convenient overview on intensity patterns. Interesting profiles can be interactively selected and mapped pathway entries inspected. Metabolite and gene entries associated with particular intensity profiles may be marked in a specific color, either by individual selection or based on previously defined labels of mapped data set features. The online resources associated with the selected pathway, e.g. the colored organism-specific KEGG pathway map, and the selected entry can be directly accessed in an additional browser window. In contrast to platforms focused on web-based interfaces (Kessler et al. 2013; Xia et al. 2012; Kastenmüller et al. 2011; Wägele et al. 2012), this approach splits the workflow into the exploratory analysis of multivariate intensity profiles by means of highly interactive desktop applications and the knowledge-based interpretation of results by means of the interconnected online resources of the KEGG and BioCyc databases. The central objective of MarVis-Pathway is the rapid detection of affected pathways that can be used as working hypotheses. This first reconstruction may be followed by a more detailed network analysis of detected pathways using specialized tools (Gao et al. 2010; Landesfeind et al. 2014; Posma et al. 2014), e.g. by means of the visualization and expansion of pathway-specific subnetworks in the Metscape software (Gao et al. 2010). In contrast to the visualization of condition-specific network snapshots, MarVis-Pathway focuses on the pathway-specific heatmap visualization of multivariate intensity profiles, which allows a convenient overview on associated intensity patterns.

### Enrichment analysis of metabolomics data sets identifies highly relevant pathways

In order to identify the most relevant pathways affected after wounding in wt and JA-deficient *dde*2-2 plants, an enrichment analysis was performed in MarVis-Pathway. First, only the four metabolomics data sets (M1–M4, see Table 1) were used for analysis. Table 2A shows the top-ranked pathways and the FDRs calculated in the entry (E-SEA), marker/feature (M-SEA), and sample-based analysis (S-SEA) (see Sects. 2.5.2 and 2.5.3). The five top-ranked pathways (see Table 2A) are highly relevant in the context of plant wounding. The jasmonic acid biosynthesis (AraCyc, rank 2) and the alpha-linolenic acid metabolism (KEGG, rank 4) pathways describe the biosynthesis of JA-Ile. Additionally, pathways associated with glucosinolate biosynthesis, which is at least in major parts regulated by JA-Ile (Sønderby et al. 2010) and which constitutes a central defense reaction of *A. thaliana* plants upon wounding, can be found in this list. For the relevant pathways, the FDRs calculated based on the M-SEA and E-SEA are much lower compared to the S-SEA. This is a direct result of the less conservative test assumptions (see Sect. 2.5.2). The data set features, e.g. different adducts of the same metabolite, or database entries, e.g. metabolites in the same pathway, are expected to show a systematic dependence (Subramanian et al. 2005; Barry et al. 2005). Nonetheless, the M-SEA is useful in order to identify pathways which contain entries that are matched by many significant features (see the jasmonic acid biosynthesis and alpha-linolenic acid metabolism pathways), indicating a correct adduct detection in preprocessing of non-targeted LC/MS data. However, this method also highlights pathways with a very low number of matched entries. The plant-pathogen interaction pathway (rank 3), that contains only one matched metabolite, JA, is an example for this case. The M-SEA and E-SEA methods require considerably less computing time in comparison to the random permutation-based S-SEA and can also be performed in case only a low number of replicate samples are available. On the other hand, the S-SEA method allows to link dependent technical replicates and samples in the random permutation test and can therefore account for dependent data sets comprising measurements for the same samples (Kaever et al. 2014). In case of the S-SEA based only on the metabolomics data sets, the estimated FDR for the important alpha-linolenic acid metabolism pathway is very high (0.807, rank 4). For most of the pathways, only a relatively small number of metabolites are matched by data set features.

**Table 2:** Top-ranked pathways from enrichment analysis based only on filtered/raw metabolomics data sets (part A), the combined metabolomics and transcriptomics data sets (B), and selected metabolomics and transcriptomics features showing a wt-constitutive intensity profile (C)

	DB	Pathway	F	M	G	M-SEA	E-SEA	S-SEA
(A) Pathway enrichment analysis of metabolomics data only								
1	KEGG	Plant hormone signal transduction	17	3	0	2.549e−06	0.005071	0.2475
2	AraCyc	Jasmonic acid biosynthesis	20	5	0	8.816e−08	0.09175	0.2475
3	KEGG	Plant–pathogen interaction	6	1	0	0.0004678	0.6159	0.357
4	KEGG	Alpha-Linolenic acid metabolism	20	13	0	2.479e−05	0.04789	0.807
5	AraCyc	Indole glucosinolate breakdown	9	4	0	0.1805	0.5675	0.8436
6	AraCyc	Heptaprenyl diphosphate biosynthesis	2	1	0	1	0.6825	0.8436
7	KEGG	Terpenoid backbone biosynthesis	2	1	0	1	1	0.8436
8	AraCyc	Glucosinolate biosynthesis from tryptophan	5	5	0	1	0.1445	0.8969
9	AraCyc	Glucosinolate biosynthesis from trihomomethionine	4	2	0	0.6825	0.9679	0.8969
10	KEGG	Sulfur relay system	2	1	0	0.2618	0.9679	0.8969
(B) Pathway enrichment analysis of metabolomics and transcriptomics data								
1	KEGG	Plant hormone signal transduction	55	3	34	2.18e−07	0.0001173	0.091
2	KEGG	Alpha-Linolenic acid metabolism	47	13	16	4.161e−18	6.228e−09	0.1363
3	KEGG	Plant–pathogen interaction	48	1	36	2.395e−09	1.13e−05	0.1363
4	AraCyc	Jasmonic acid biosynthesis	43	5	14	1.515e−15	0.0002319	0.1363
5	KEGG	Glucosinolate biosynthesis	24	12	9	0.0008703	1.001e−05	0.1578
6	KEGG	Fatty acid elongation	11	0	9	0.02568	0.01554	0.2113
7	AraCyc	Hydroxyjasmonate sulfate biosynthesis	3	0	2	0.01398	0.1264	0.2113
8	KEGG	Carotenoid biosynthesis	9	1	6	0.3106	0.341	0.3406
9	AraCyc	traumatin and (Z)-3-hexen-1-yl acetate biosynthesis	13	0	6	2.783e−06	0.08044	0.4552
10	AraCyc	Glucosinolate biosynthesis from tryptophan	15	5	9	0.01398	0.0005411	0.5308
(C) Pathway enrichment analysis for selected wt-constitutive features								
1	AraCyc	Glucosinolate biosynthesis from tryptophan	5	5	0	0.02223	0.001129	–
2	AraCyc	Sulfate activation for sulfonation	2	0	2	0.002438	0.006558	–
3	KEGG	Tryptophan metabolism	5	3	1	0.1026	0.01164	–
4	KEGG	Glucosinolate biosynthesis	5	5	0	0.1088	0.02593	–
5	KEGG	Sulfur metabolism	2	0	2	0.01791	0.02744	–
6	KEGG	2-Oxocarboxylic acid metabolism	5	5	0	0.3276	0.1019	–
7	KEGG	Purine metabolism	2	0	2	0.1026	0.2686	–
8	AraCyc	Glucosinolate biosynthesis from homomethionine	2	1	1	0.3276	0.41	–
9	AraCyc	Glucosinolate breakdown	1	0	1	0.1672	0.4173	–
10	KEGG	Stilbenoid, diarylheptanoid and gingerol biosynthesis	2	2	0	0.4627	0.4173	–

The 4th, 5th, and 6th column contain the number of filtered/selected features over all data sets (F) which could be assigned to an entry in the corresponding pathway, the number of matched metabolites (M) in the corresponding pathway, and the number of matched genes (G). The last columns contain the estimated false discovery rates (FDRs) based on a marker/feature-based SEA (M-SEA), entry-based SEA (E-SEA), and sample-based SEA (S-SEA). The pathways are sorted according to the S-SEA (A, B) or E-SEA FDRs (C), respectively

### Transcriptomics data significantly support the pathway analysis

**Fig. 4:**
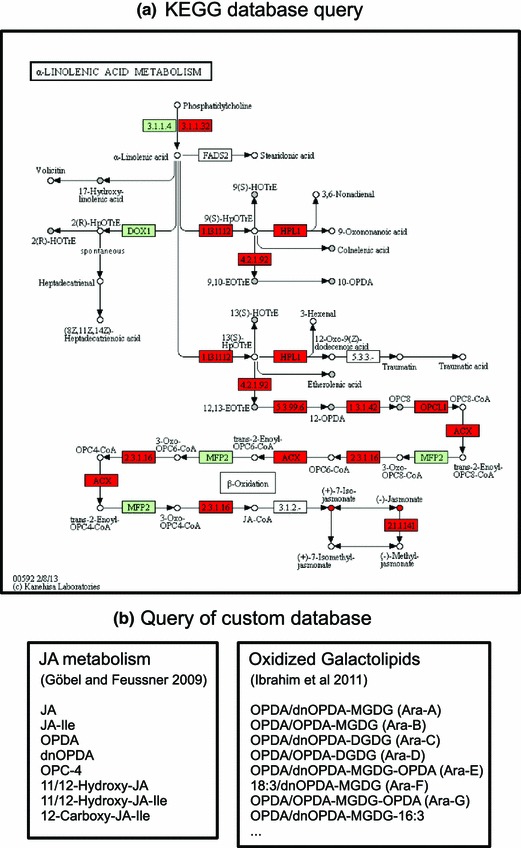
Results from database query in MarVis-Pathway. **a** The KEGG alpha-linolenic acid metabolism pathway with entries mapped to features from the filtered metabolomics and transcriptomics data sets. Entries exclusively mapped to labeled features, which are specific for the wounding of wt plants, are marked in *red*. Entries mapped to features which are not associated with a wt-specific intensity profile, e.g. because of the mapping of isomers with different intensity patterns to the same metabolite, are marked in *gray*. *Green color* indicates enzymes associated with *A. thaliana* genes which could not be mapped to features from the filtered transcriptomics data set. **b** Wt-specific feature hits from the query of a custom database containing metabolites from the jasmonic acid (JA) metabolism and oxidized galactolipids described in literature. *10-OPDA* 10-oxo-11,15-phytodienoic acid, *12-OPDA* 12-oxo-10,15-phytodienoic acid, *9,10-EOTrE* 9,10-epoxyoctadecatrienoic acid, *12,13-EOTrE* 12,13-epoxyoctadecatrienoic acid, *OPC-8:0* 3-oxo-2-(pent-2’-enyl)-cyclopentane-1-octanoic acid, *9(S)-HOTrE* 9-hydroxyoctadecatri-10,12,15-enoic acid, *13(S)-HOTrE* 13-hydroxyoctadeca-9,11,15-trienoic acid, *2(R)-HOTrE* 2-hydroxyoctadecatri-9,12,15-enoic acid, *JA-Ile* jasmonoyl isoleucine, *dnOPDA* 10-oxo-8,13-dinor-phytodienoic acid, *OPC-4* 3-oxo-2-(pent-2′-enyl)-cyclopentane-1-butanoic acid, *DGDG* digalactosyl diacylglycerol, *MGDG* monogalactosyl diacylglycerol

The pathway enrichment analysis was repeated for the metabolomics (M1–M4) in combination with the transcriptomics (T1) data set. For the S-SEA, the sample labels in the metabolomics and transcriptomics data sets were linked (see Sect. 2.5.3). The enrichment analysis (see Table 2B) results in much lower estimated FDRs compared to the case where only the metabolomics data sets were used (see Table 2A). Especially the E-SEA method is highly sensitive to the higher coverage of database entries due to the assigned transcript features (see alpha-linolenic acid metabolism pathway, rank 2). The alpha-linolenic acid metabolism pathway is also associated with a much lower FDR for the S-SEA method (0.1363) compared to the FDR estimated without the microarray data set (0.807). Figure 4a shows the corresponding colored KEGG pathway map. Entries (metabolites and genes) mapped to data set features which were labeled as specific for the wounding of wt plants are marked in red. Entries mapped to features which are not associated with a wt-specific intensity profile are marked in gray. This pathway, which describes the jasmonate biosynthesis and contains the allene oxide synthase (AOS) enzyme (EC 4.2.1.92) that is missing in the *dde*2-2 mutant, should be highly enriched in features showing significant differences between the experimental conditions and especially features with a wt-specific profile. From the metabolomics point of view, only the jasmonate is clearly associated with wt-specific ion features. All other matched metabolites (gray points) are not exclusively associated with labeled features due to isomers and ambiguous mass matching (see mapping table in Supplementary material 1). By means of the mapping of the filtered microarray data set, the coverage of pathway entries is significantly increased, as represented by much lower FDRs in enrichment analysis, and the wt-specific enzymatic steps towards the biosynthesis of jasmonate are clearly highlighted (see the lower branch of the pathway). Noteably, all but two mapped transcript features are labeled as wt-specific (see mapping table in Supplementary material 1).

The integration of the transcriptomics data set has a strong effect on the estimated FDRs. However, the microarray data do not bias the overall pathway ranking. In both cases, when analyzing only the metabolomics (see Table 2A) or transcriptomics data (see Supplementary material 7), the highly relevant alpha-linolenic acid metabolism, the plant hormone signal transduction, and glucosinolate-related pathways can be found in the list of top-ranked candidates. In addition, the introduced methods for integrative enrichment and meta-analysis do not depend on the estimation of a time lag between data from different omics platforms (Takahashi et al. 2011). The introduced analysis is based on the ranking of data set features according to general differences between the experimental conditions or the selection of features associated with particular intensity patterns.

### Custom databases expand pathway analysis

The analysis based on KEGG and AraCyc pathways resulted in a relatively small number of metabolite annotations (see Table 2A) because many precursors and derivatives of jasmonic acid as well as related compound classes, such as oxidized galactolipids, are not yet represented in these databases. In order to integrate expert and literature knowledge, MarVis-Pathway provides an interface to import custom databases in CSV format, containing additional entries (e.g. metabolites, genes, or enzymes) and assignments to pathways or arbitrary sets/groups of related entries, such as compound classes (see workflow in Fig. 1 and MarVis-Suite handbook). For data analysis in this study on plant wounding, a custom database containing previously described metabolites (Göbel and Feussner 2009; Ibrahim et al. 2011) was created (see custom database in Supplementary material 2). This database was used for annotating additional metabolic features based on the corrected masses (see Fig. 4b and the table of additional metabolite hits in Supplementary material 3). By this means, 22 highly context-related metabolites could be assigned to features which exclusively accumulated in wt plants after wounding. These JA-Ile-dependent wound-induced features are represented by prototypes 1 to 6 after clustering by 1D-SOM (see Fig. 2). As proof of concept, five putative metabolite hits, including JA and JA-Ile as well as the JA-derivatives described as degradation products or transport forms, 12-hydroxy-JA, 12-hydroxy-JA-Ile, and 12-carboxy-JA-Ile, were confirmed by MS/MS analysis (see MS/MS spectra in Supplementary material 4).

In the following, we will describe two further examples how the new MarVis-Suite tools support the exploratory analysis and context-related identification of data set features.

### Pathway analysis of selected clusters identifies glucosinolates as JA-Ile-dependent metabolites with wt-constitutive intensity pattern

The prototype heatmap for the combined cross-omics data set (see Fig. 2) shows a number of other interesting intensity patterns. For example, cluster 10 contains features with a wt-constitutive pattern characterized by very small differences between the wt conditions and zero or very low average intensities for the mutant-associated conditions. For further analysis, the cluster was selected in MarVis-Cluster and only the associated features were imported and analyzed in MarVis-Pathway. Table 2C shows the results of marker and entry-based enrichment analysis. Interestingly, most of the top-ranked pathways are associated with glucosinolate biosynthesis (see mapping table in Supplementary material 5). Though, only a small number of features match entries in these pathways.

### Customized SNR ranking detects *dde*2-2-constitutive intensity profiles

In contrast to the wt-constitutive intensity pattern, the prototype heatmap (see Fig. 2) does not reveal intensity profiles with a *dde*2-2-constitutive pattern. However, there may be a small number of corresponding features hidden in one of the more prominent clusters. Therefore, the whole cross-omics data set was re-ranked in MarVis-Filter utilizing a signal-to-noise ratio with customized signal term (see Sect. 2.3), the difference between the minimum over the average intensities of the *dde*2-2-associated conditions 4–6 and the maximum over the average intensities of the wt-associated conditions 1–3. Interestingly, only two of the 2,809 filtered transcriptomics data set features, ambiguously associated with At1g53490 and At1g53480, could be found with a ratio greater than 2 (see expression profiles in Supplementary material 6). These two microarray spots show high expression levels for the *dde*2-2-associated conditions independent of the wounding and may be an interesting starting point for further studies on the *dde*2-2 mutant.

## Concluding remarks

The MarVis-Suite combines a statistical framework with highly interactive interfaces for exploratory data analysis. Data sets from different omics platforms can be filtered, combined, clustered, and visualized. By means of the new MarVis-Pathway interface, filtered or selected data set features may be annotated in the context of organism-specific pathway databases or custom pathway/entry set definitions which represent expert knowledge. The signal-to-noise ratio allows the ranking and filtering of heterogeneous data sets within a common framework and can easily be customized for the search for particular intensity patterns. The framework allows many other options, including alternative ratios, e.g. the signal-to-level ratio, or moderation/shrinkage of the noise term (Smyth 2004; Allison et al. 2006). By means of the enrichment analysis, annotated pathways can be statistically evaluated based on different assumptions, e.g. independence of features, database entries, or samples. Additionally, MarVis-Pathway provides functions for the meta-analysis of pathway enrichment for multiple data sets. The tools were successfully applied in a cross-omics study on plant wounding. The integration of transcriptomics data significantly supported the analysis of the non-targeted metabolomics data sets. Additionally, proteomics data can be integrated for a more comprehensive analysis.

## Electronic supplementary material

Below is the link to the electronic supplementary material.

Supplementary material 1 (XLS 38 kb)

Supplementary material 2 (CSV 19 kb)

Supplementary material 3 (XLS 31 kb)

Supplementary material 4 (PDF 258 kb)

Supplementary material 5 (XLS 8 kb)

Supplementary material 6 (TIFF 1,334 kb)

Supplementary material 7 (PDF 35 kb)
