# Supplementary material for: MarVis-Pathway: integrative and exploratory pathway analysis of non-targeted metabolomics data
Source: Metabolomics. 2014 Oct 10;11(3):764–77. doi: 10.1007/s11306-014-0734-y (PMC4419191; doi:10.1007/s11306-014-0734-y)

Jasmonic acid (JA)

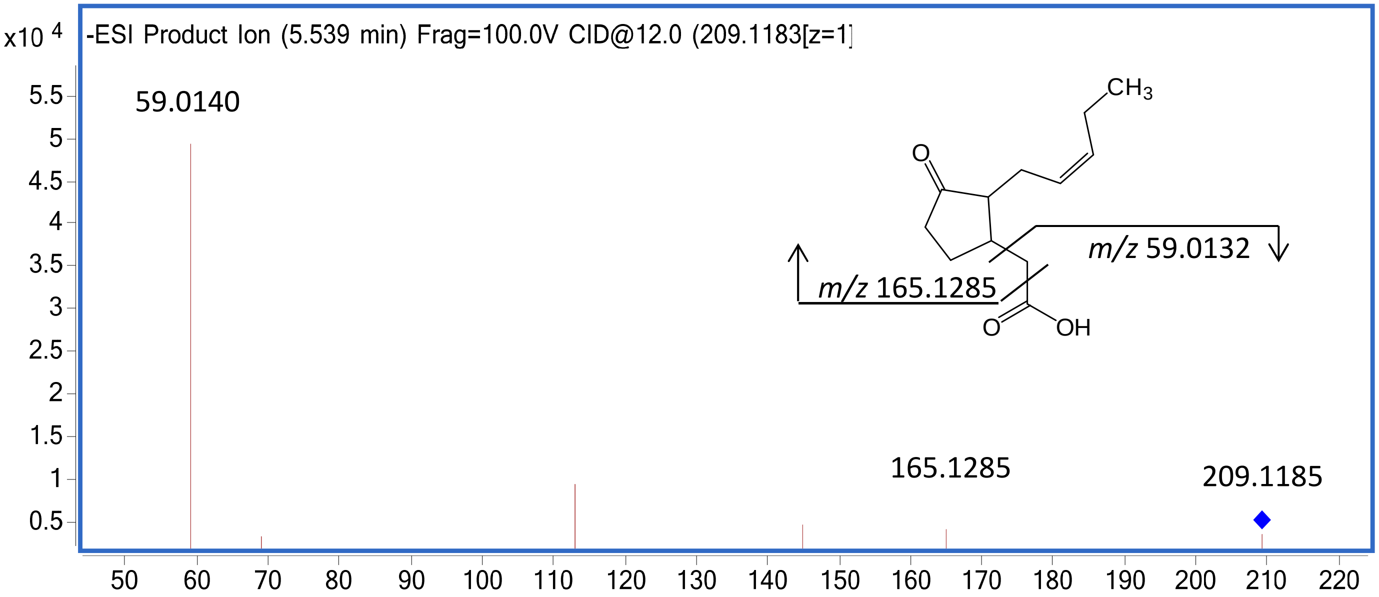

Jasmonoyl isoleucine (JA-Ile)

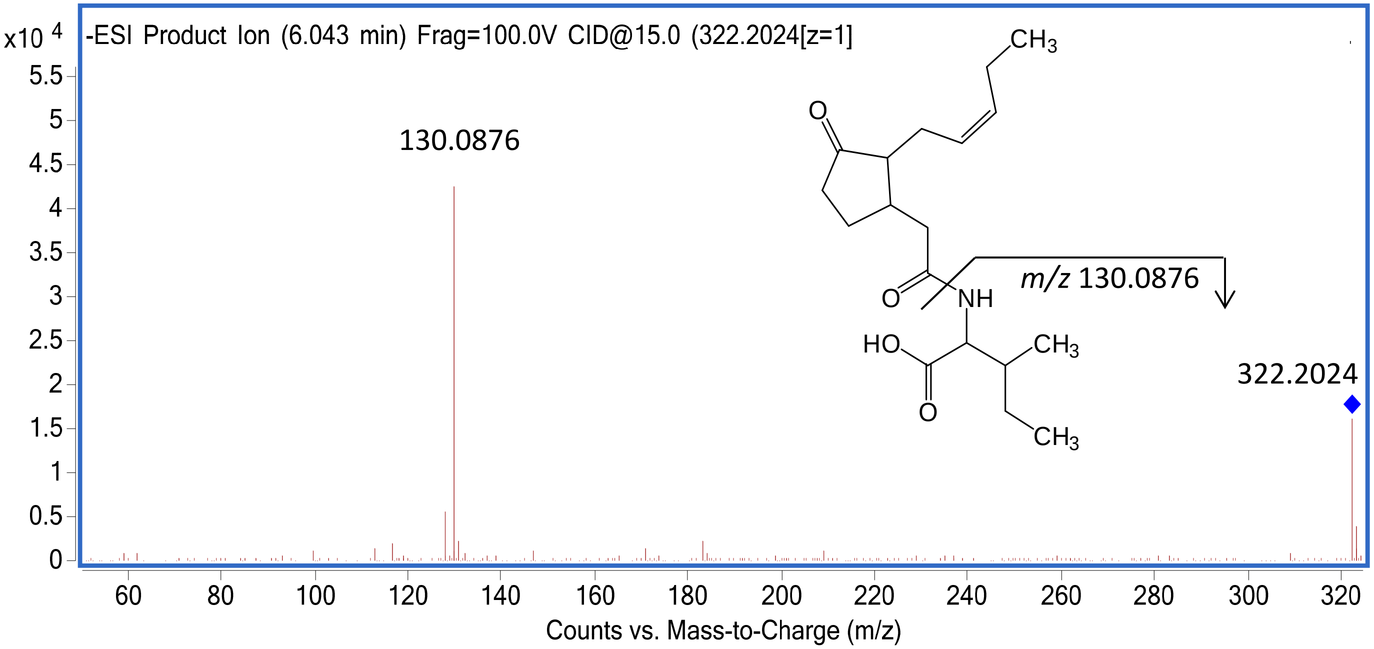

12-Carboxyjasmonoyl-L-isoleucine (12-Carboxy-JA-Ile)

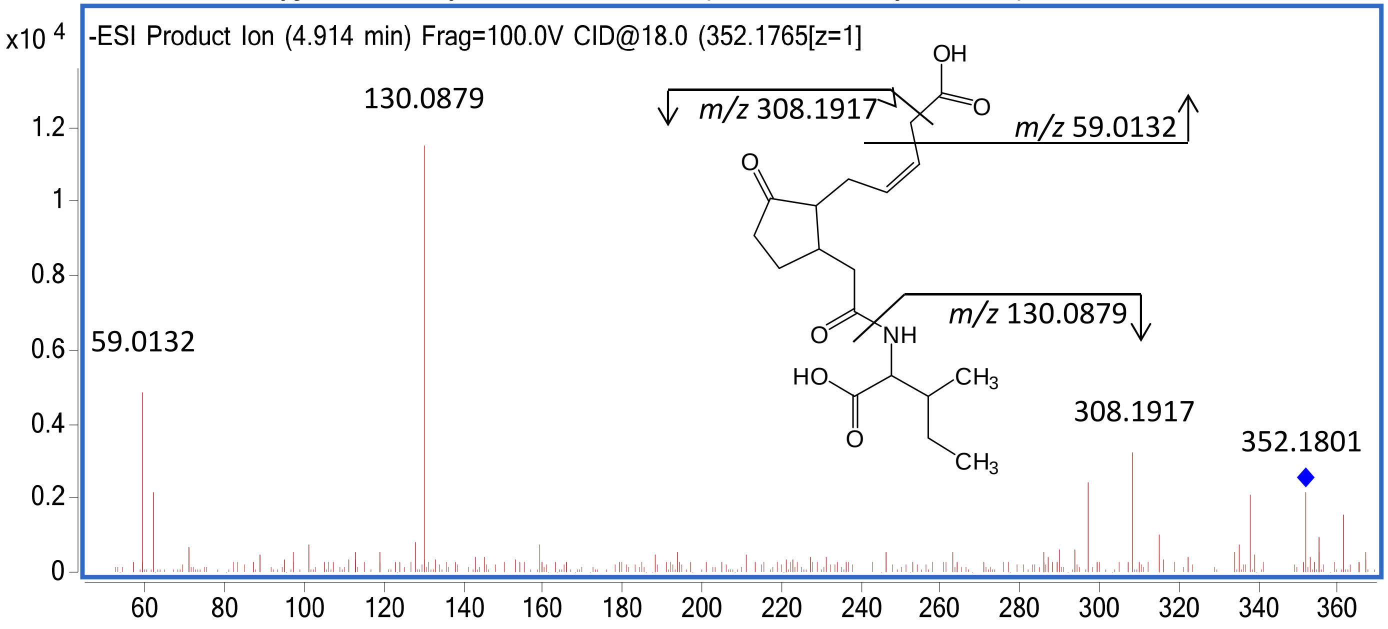

12-Hydroxy jasmonoyl isoleucine (12-Hydroxy-JA-Ile)

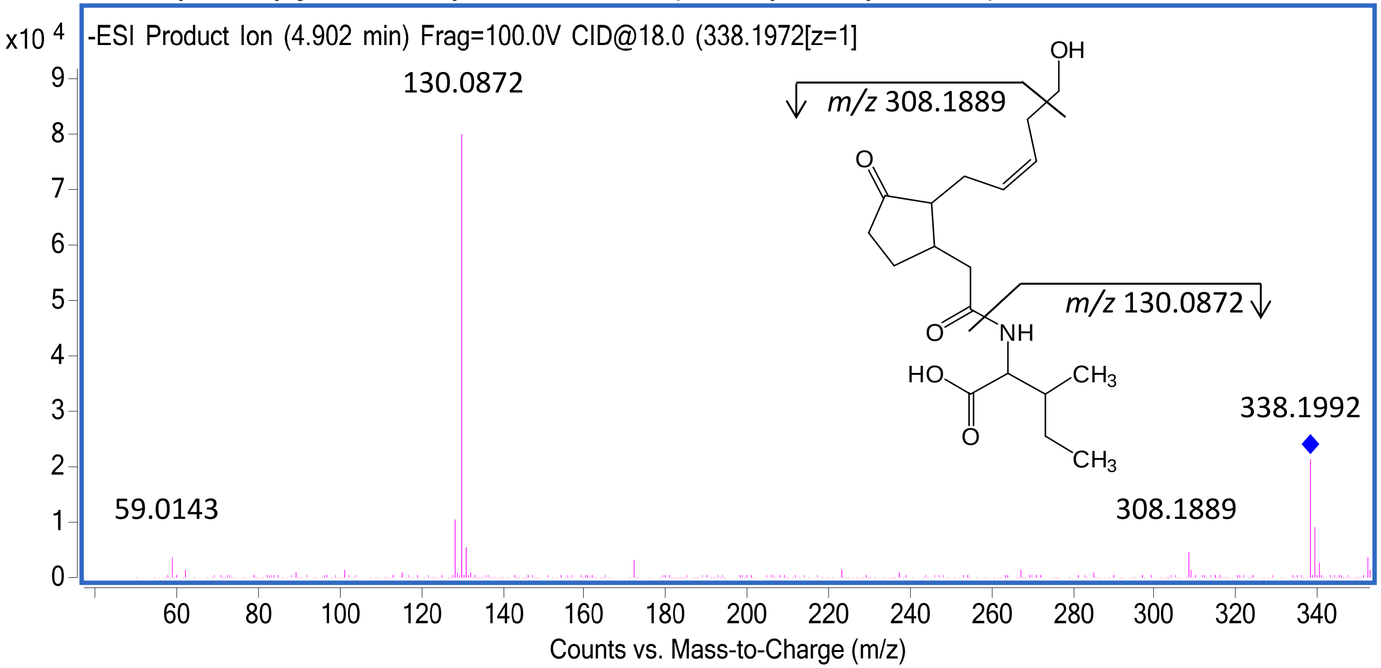

12-Hydroxy jasmonic acid (12-Hydroxy-JA)

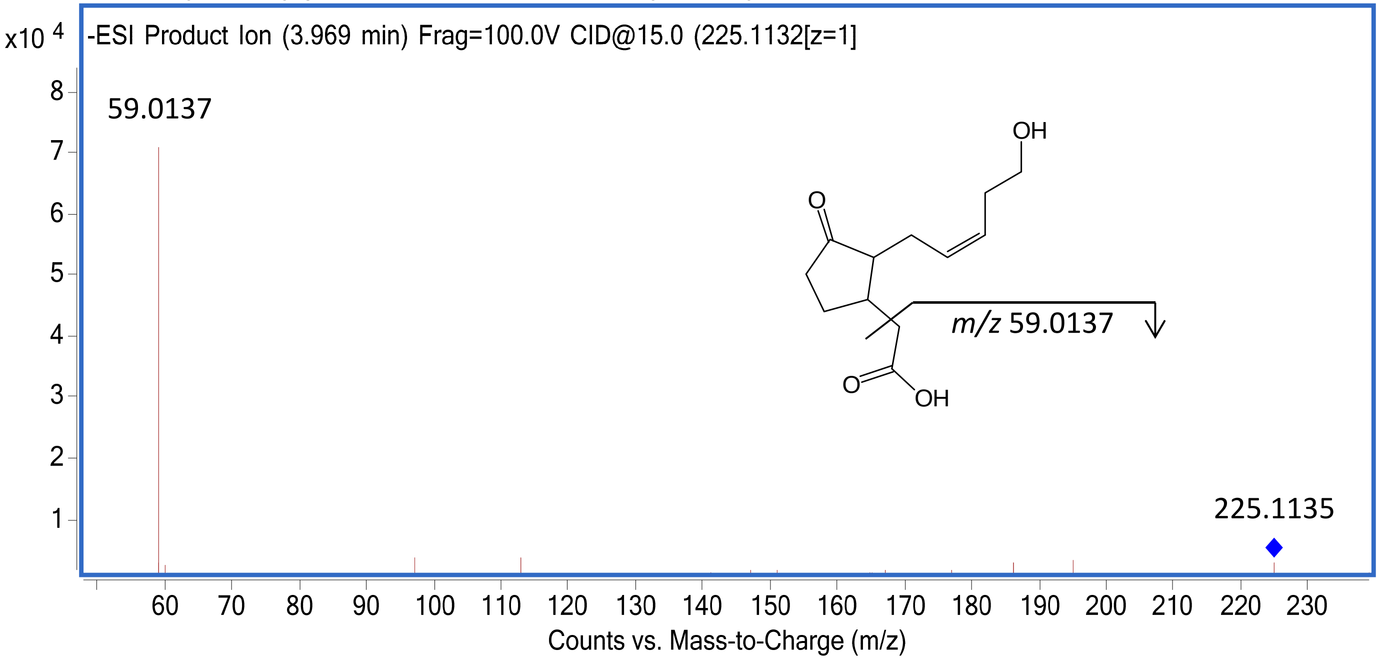

Supplement: Supplementary file 4 — Supplementary material 4 (PDF 258 kb) [file 11306_2014_734_MOESM4_ESM.pdf]
