# Supplementary figures and images for: MarVis-Pathway: integrative and exploratory pathway analysis of non-targeted metabolomics data
Source: Metabolomics. 2014 Oct 10;11(3):764–77. doi: 10.1007/s11306-014-0734-y (PMC4419191; doi:10.1007/s11306-014-0734-y)

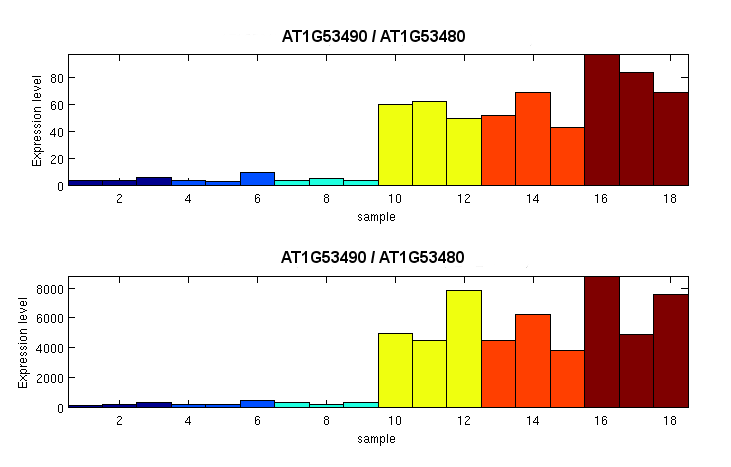

Supplement: Supplementary file 6 — Supplementary material 6 (TIFF 1,334 kb) [file 11306_2014_734_MOESM6_ESM.tiff]
