# Supplementary material for: MarVis-Pathway: integrative and exploratory pathway analysis of non-targeted metabolomics data
Source: Metabolomics. 2014 Oct 10;11(3):764–77. doi: 10.1007/s11306-014-0734-y (PMC4419191; doi:10.1007/s11306-014-0734-y)
Supplement: Supplementary file 7 — Supplementary material 7 (PDF 35 kb) [file 11306_2014_734_MOESM7_ESM.pdf]

Table 1: Top-ranked pathways from enrichment analysis of the filtered/raw Transcriptomics data set. The 4th, 5th, and 6th column contain the number of filtered/selected features (F) which could be assigned to an entry in the corresponding pathway, the number of matched metabolites (M) in the corresponding pathway, and the number of matched genes (G). The last columns contain the estimated false discovery rates (FDRs) based on a marker/feature-based SEA (M-SEA), entry-based SEA (E-SEA), and sample-based SEA (S-SEA). The pathways are sorted according to the S-SEA FDRs.

**Pathway enrichment analysis of Transcriptomics data**

| | DB | Pathway | F | M | G | M-SEA | E-SEA | S-SEA |
|---|---|---|---|---|---|---|---|---|
| 1 | KEGG | Glucosinolate biosynthesis | 12 | 0 | 9 | 4.936e-05 | 0.0009647 | 0.1373 |
| 2 | AraCyc | hydroxyjasmonate sulfate bio-synthesis | 3 | 0 | 2 | 0.01173 | 0.1399 | 0.1373 |
| 3 | KEGG | alpha-Linolenic acid metabolism | 27 | 0 | 16 | 2.778e-13 | 1.523e-07 | 0.1373 |
| 4 | AraCyc | traumatin and (Z)-3-hexen-1-yl acetate biosynthesis | 13 | 0 | 6 | 3.287e-06 | 0.08067 | 0.1373 |
| 5 | KEGG | Fatty acid elongation | 11 | 0 | 9 | 0.02166 | 0.01983 | 0.1373 |
| 6 | KEGG | Plant hormone signal transduction | 38 | 0 | 34 | 0.1314 | 0.1399 | 0.1373 |
| 7 | KEGG | Phenylalanine, tyrosine and tryptophan biosynthesis | 22 | 0 | 17 | 8.786e-07 | 0.0003148 | 0.1373 |
| 8 | AraCyc | very long chain fatty acid biosynthesis I | 6 | 0 | 4 | 0.4122 | 0.921 | 0.1658 |
| 9 | AraCyc | superpathway of phenylalanine, tyrosine and tryptophan biosynthesis | 16 | 0 | 13 | 9.035e-05 | 0.1359 | 0.1658 |
| 10 | AraCyc | tryptophan biosynthesis | 11 | 0 | 8 | 3.266e-05 | 0.06561 | 0.1658 |
